# Dysregulation of MicroRNAs and Target Genes Networks in Peripheral Blood of Patients With Sporadic Amyotrophic Lateral Sclerosis

**DOI:** 10.3389/fnmol.2018.00288

**Published:** 2018-08-28

**Authors:** Maria Liguori, Nicoletta Nuzziello, Alessandro Introna, Arianna Consiglio, Flavio Licciulli, Eustachio D’Errico, Antonio Scarafino, Eugenio Distaso, Isabella L. Simone

**Affiliations:** ^1^National Research Council, Institute of Biomedical Technologies, Bari Unit, Bari, Italy; ^2^Department of Basic Sciences, Neurosciences and Sense Organs, University of Bari, Bari, Italy

**Keywords:** sporadic amyotrophic lateral sclerosis, microRNA, target genes, peripheral blood markers, high throughput next-generation sequencing (HT-NGS), clinical parameters, bioinformatics, pathway analysis

## Abstract

Amyotrophic lateral sclerosis (ALS) is a progressive and fatal neurodegenerative disease. While genetics and other factors contribute to ALS pathogenesis, critical knowledge is still missing and validated biomarkers for monitoring the disease activity have not yet been identified. To address those aspects we carried out this study with the primary aim of identifying possible miRNAs/mRNAs dysregulation associated with the sporadic form of the disease (sALS). Additionally, we explored miRNAs as modulating factors of the observed clinical features. Study included 56 sALS and 20 healthy controls (HCs). We analyzed the peripheral blood samples of sALS patients and HCs with a high-throughput next-generation sequencing followed by an integrated bioinformatics/biostatistics analysis. Results showed that 38 miRNAs (let-7a-5p, let-7d-5p, let-7f-5p, let-7g-5p, let-7i-5p, miR-103a-3p, miR-106b-3p, miR-128-3p, miR-130a-3p, miR-130b-3p, miR-144-5p, miR-148a-3p, miR-148b-3p, miR-15a-5p, miR-15b-5p, miR-151a-5p, miR-151b, miR-16-5p, miR-182-5p, miR-183-5p, miR-186-5p, miR-22-3p, miR-221-3p, miR-223-3p, miR-23a-3p, miR-26a-5p, miR-26b-5p, miR-27b-3p, miR-28-3p, miR-30b-5p, miR-30c-5p, miR-342-3p, miR-425-5p, miR-451a, miR-532-5p, miR-550a-3p, miR-584-5p, miR-93-5p) were significantly downregulated in sALS. We also found that different miRNAs profiles characterized the bulbar/spinal onset and the progression rate. This observation supports the hypothesis that miRNAs may impact the phenotypic expression of the disease. Genes known to be associated with ALS (e.g., *PARK7, C9orf72, ALS2, MATR3, SPG11, ATXN2*) were confirmed to be dysregulated in our study. We also identified other potential candidate genes like *LGALS3* (implicated in neuroinflammation) and *PRKCD* (activated in mitochondrial-induced apoptosis). Some of the downregulated genes are involved in molecular bindings to ions (i.e., metals, zinc, magnesium) and in ions-related functions. The genes that we found upregulated were involved in the immune response, oxidation–reduction, and apoptosis. These findings may have important implication for the monitoring, e.g., of sALS progression and therefore represent a significant advance in the elucidation of the disease’s underlying molecular mechanisms. The extensive multidisciplinary approach we applied in this study was critically important for its success, especially in complex disorders such as sALS, wherein access to genetic background is a major limitation.

## Introduction

Amyotrophic lateral sclerosis is a progressive neurodegenerative disease in which different pathogenic mechanisms, including inflammation, oxidative stress, glutamate excitotoxicity, protein misfolding, apoptosis and dysfunction of axonal transport, have been identified (Al-Chalabi et al., 2017; Vejux et al., 2018). Clinically, the disease predominantly affects upper and lower motor neurons, although impairment of extramotor systems – such as temporal, behavioral and executive frontal circuits – has also been reported suggesting that ALS should be better considered a multisystem disorder (Phukan et al., 2007; Goldstein and Abrahams, 2013). Progressive spinal muscular atrophy and primary lateral sclerosis have been classified as restricted phenotypes of ALS (10% of cases) but a clear separation is still controversial (Gordon et al., 2013). Isolated bulbar involvement (5%) and association with cognitive/behavioral signs that fulfill the diagnostic criteria of frontotemporal dementia (FTD) (5–15%) (Strong et al., 2017) underline the heterogeneity of the disease that also involves the age at onset, the rate of progression and finally the overall prognosis, thus drawing a complex scenario of the ALS phenotypes (Swinnen and Robberecht, 2014).

Most ALS cases are sporadic (sALS), whereas a family history (fALS) is found in 10% of patients. Indeed, several factors other than genes (toxic exposures, diet, and others) seem to possibly contribute to ALS pathogenesis (Paez-Colasante et al., 2015; Morgan and Orrell, 2016; Al-Chalabi et al., 2017) but a definitive conclusion on the effective role of the different factors is still awaited, mostly due to methodological biases (i.e., low power of the studies) (van Es et al., 2017).

Another unsolved issue is the lack of validated biomarkers for ALS pathogenesis and monitoring, mainly due to the phenotypic heterogeneity of the disease. Molecular markers have been first searched in the CSF, since liquor may provide more biological information on the biochemical and molecular processes underlying the disease, given its intimate connection with the central nervous system (CNS). Indeed, suggestive CSF markers have been evaluated over the years, with particular attention to those related to neuroinflammation, like metalloproteinases-2 and -9, IL-2 and -6, or neurodegeneration, e.g., Tau-protein or TDP-43 (Vejux et al., 2018). However, presently only the neurofilament (NF), main product of neuroaxonal breakdown, seems to represent a sensitive biomarker of neurodegeneration (Lu et al., 2012). Significant increase of NF was found in the CSF and serum of ALS patients, thus justifying its inclusion in the diagnostic protocol as well as in the evaluation of clinical course, given an increased level of NF-light chain in CSF of ALS that was found to be predictive of rapid clinical progression (Tortelli et al., 2015; Steinacker et al., 2016). Recently, a report showed increased levels of three macrophage-derived chitinases (CHIT1, CHI3L1, and CH13L2) in CSF of ALS patients and found a correlation with the progression rate of the disease, independently of their NF levels (Thompson et al., 2018). However, lately the search for biomarkers has increasingly focused on blood samples (i.e., serum and plasma) obtained from a less invasive procedure, in an effort to identify early markers of ALS onset as well as those useful for monitoring its progression (Vu and Bowser, 2017).

The report on the implication of two ALS genes, TDP-43 and FUS, in the biogenesis of microRNAs (miRNAs) has sparked great interest about their potential role in the pathogenesis and progression of ALS (Freischmidt et al., 2013). miRNAs are small non-coding RNA molecules that regulate at the post-transcriptional level the expression of genes involved in cellular response to stressors and other pathogenic insults (Viader et al., 2011; Kye and Goncalves Ido, 2014). Interestingly, despite being found rapidly degraded in post-mortem brain tissue, miRNAs are stable in serum and other body fluids, such as CSF (Saraiva et al., 2017). Therefore their evaluation can be informative in healthy as in pathological conditions. In ALS, several studies have reported the occurrence of miRNAs dysregulation (Waller et al., 2017b; Matamala et al., 2018; Vejux et al., 2018; Vrabec et al., 2018). Among the others, a disease-specific two-fold upregulation of miR-338-3p, involved in apoptosis, neurodegeneration and glutamate clearance, has been reported in blood leukocytes, CSF serum, and spinal cords of patients with sALS compared to controls (De Felice et al., 2014). Furthermore, 30 miRNAs significantly downregulated have been identified in the serum of fALS patients, the majority of them already dysregulated in pre-symptomatic subjects carrying some of the mutations causative of the disease (Freischmidt et al., 2014). These miRNAs represent potential targets for therapeutic interventions at the very early stages of the disease.

The availability of high-throughput technologies for large scale sequencing (HT-NGS) significantly improved the possibility of investigating with an unbiased approach the patterns of miRNAs associated with diseases like ALS. Importantly, miRNAs expression profiles can reflect the activation of specific pathogenic pathways in many neurodegenerative diseases, including ALS (Waller et al., 2017a). That approach, in combination with the evaluation of genes expression (i.e., mRNA), could reveal novel pathogenic hypotheses worthy of testing.

Based on the above developments we performed an extensive transcriptomic investigation in sALS patients with the primary aim of identifying dysregulation of miRNAs and mRNAs associated with the disease, and secondly to analyze their possible role as modulating factors of clinical features.

## Materials and Methods

We planned a multidisciplinary strategy starting with the subjects’ selection, the molecular analyses on their peripheral blood samples, as well as the bioinformatics/statistics evaluations of the data.

### Subject Recruitment and Clinical Evaluation

Patients with probable or definite ALS (revised El Escorial Criteria) (Brooks et al., 2000) were recruited at the time of their first diagnosis (age ranging from 18 to 80 years) at the Department of Basic Sciences, Neurosciences and Sense Organs, University of Bari, Bari, Italy. Subjects with positive history of other neurological diseases, head/spinal trauma, psychiatric disorders, and alcohol/psychotropic drug use were excluded from the study. The search for mutations within those genes (and flanking intron–exon boundaries) that are commonly associated with ALS in our geographic area (Southern Italy) returned negative for all except six patients (two with SOD1 mutation, one patient with TARDBP mutation and three carrying the pathogenic C9orf72 expansions). Since no familial history of ALS was ascertained at the study entry, they were considered *de novo* mutations or mutations with incomplete penetrance.

We divided the study in two phases. In the first one (*discovery phase*) a small sample of sALS patients was recruited, whereas in the second phase (*validation phase*) we examined a larger and distinct sample of sALS patients. We collected the following information from all patients: demographic and clinical data including gender, age at symptom onset as referred by the patient, site of onset (bulbar or spinal), ODI (onset to diagnosis interval), time to generalization (time interval between disease spreading from spinal to bulbar district or *vice versa*), disease duration (time interval between symptom onset and blood sampling). Clinical severity was assessed by the revised ALS Functional Rating scale (ALSFRSr) (Cedarbaum et al., 1999) and the Manual Muscle Testing (MMT) (medium score). Disease progression rate was calculated as: (48-ALSFRSr score at blood sampling)/disease duration at blood sampling. Forced vital capacity was also measured. The enrollment end-date was December 31, 2017.

We recruited healthy subjects with no history of neurological diseases as HCs in the same geographic area (Bari, Southern Italy).

The Ethic Committee of Azienda Ospedaliera Policlinico, University of Bari, Italy, approved the study and we obtained a signed informed consent from all participants at the time of their enrollment (according to the Declaration of Helsinki^1^).

### Molecular Analysis

Peripheral blood samples were taken from patients and controls and stored at -20°C in 3 ml PAXgene Blood RNA Tubes (PreAnalytiX Qiagen/BD, Hombrechtikon, Switzerland). Total RNA was isolated using the PAXgene Blood RNA Kit (PreAnalytiX Qiagen/BD, Hilden, Germany) at the Institute of Biomedical Technologies, National Research Council, Bari, Italy. RNA concentration and purity were measured by Nanodrop ND-1000 (Thermo Scientific, Wilmington, DE, United States) and RNA 6000 Pico chip on Bioanalyzer 2100 (Agilent Technologies, Santa Clara, CA, United States), respectively. Samples with RNA integrity number (RIN) scores higher than 7 and with A260/A280 values in the 1.8–2.2 range were processed for deep sequencing.

### HT-NGS (Discovery Phase)

The RNA samples were sequenced using an Illumina HiSeq2500 platform. SmallRNA (sRNA) libraries were prepared by the TruSeq sRNA Sample Preparation kit (Illumina) and their quality was confirmed on a Bioanalyzer 2100 instrument. A multiplexed pool of equimolar amounts of individual sRNA-derived libraries was sequenced to generate 50 bp single-end reads, resulting in around 10 million reads/sample. The mRNA libraries were prepared using the TruSeq Stranded mRNA Sample Preparation kit, fluorimetrically quantified and analyzed, pooled together to obtain equimolar concentrations into a multiplex sequencing pool and sequenced to generate 2 bp × 100 bp paired-end reads (around 30 million reads/sample).

### RT and Microfluidic_qPCR (Validation Phase)

Total RNA/sample was reverse transcribed into cDNA, amplified, diluted, and used as a template for microfluidic_qPCR analysis (TaqMan Advanced miRNA Cards, ABI). PCR amplification was performed under the manufacturer’s protocols. Raw_Ct-values were calculated using Expression Suite^TM^ software v1.1 (Life Technologies, Thermo Fisher Scientific). The auto-baseline algorithm in the software was used to compensate for background noise for each amplification curve, and the thresholds were automatically adjusted to the log-linear range (Liguori et al., 2018).

### HT-NGS Data Analysis

We processed sRNA/RNA-Seq data according to a bioinformatics pipeline that we developed and tested in other neurological diseases (Liguori et al., 2018) and that consisted in the steps described below.

#### Quality Check

The quality control of the obtained reads was performed using the FastQC package^2^. We checked for low-quality reads by base sequence quality, sequence quality scores, base sequence content, base GC content, sequence GC content, base N content, sequence length distribution, sequence duplication levels, overrepresented sequences, and kmer content. If reads were of low quality by the above criteria, we removed them from the subsequent analysis.

#### Read Identification (sRNA)

The sRNA reads were mapped, using Bowtie aligner (Langmead et al., 2009), an ultrafast and memory-efficient alignment of short DNA sequences to the human genome, against an *in-house-developed* reference database ncRNAdb, a comprehensive and non-redundant dataset of non-coding (nc-RNA) sequences and annotations extracted from public database like miRBase^3^, Vega^4^, Ensembl^5^, RefSeq^6^, piRNAbank^7^, GtRNAdb^8^, and HGNC^9^. The reads that were not mapped to known ncRNAs were aligned against the human genome and passed to mirDeep2 software^10^, which computationally identifies novel miRNA and their mature miRNA products.

#### Read Identification (mRNA)

The reads obtained from total RNA were mapped against the human genome and known human transcripts (GRCh38), using Bowtie2 which supports gapped alignment and is faster on long paired-end reads.

#### Expression Quantification

In order to obtain reliable read counts and to fix the problem of multireads (reads mapping to more than one reference location) (Consiglio et al., 2016), we employed the RSEM tool for accurate expression estimations (Li and Dewey, 2011). The count values produced by the Bayesian model implemented in RSEM were used as expression values in this work. When normalization of the expressions was necessary for some analysis steps, the trimmed mean of M-values (TMM) normalization method was used (Robinson and Oshlack, 2010).

#### Differential Expression (DE) Analysis

Expression estimations computed for mRNAs (coding genes) and small ncRNAs were compared among the sALS and HC groups with the aim of determining statistically significant changes in the levels of expression. Since this is a very crucial step in the bioinformatics workflow and there is no general consensus regarding which method performs best in a given situation, we combined the results of three different software packages for DE analysis: edgeR^11^, the DESeq2^12^, and the limma^13^. The edgeR and DESeq2 were designed for NGS data and include data normalization and *p*-value correction for multiple testing by false discovery rate (FDR). The limma software was recently upgraded to enable measurements from read counts while taking into account the peculiarities of RNA-seq data (Law et al., 2014). Specifically, genes were filtered out if they failed to achieve a count per million (cpm) value of 1 in at least 20% of samples. The expressions were simultaneously scale normalized using TMM and variance was stabilized using the voom technique. The corresponding log-cpm values and associated weights were the inputs in the limma standard linear modeling and empirical Bayes for DE analysis. The change in the expression was considered statistically significant if the adjusted *p*-value was < 0.05.

### Statistical Analysis

#### For qRT-PCR Data

Two normalization tools, NormFinder and geNorm, were used to identify the most suitable endogenous reference genes. The comparison of normalized values between the subgroups was obtained according to the 2^-ΔΔCt^ method (*p*-value < 0.05). Normality of data was assessed by the Shapiro–Wilk test. We performed the statistical analysis between miRNAs (DE) with the Expression Suite software, which consists of two-tailed Student’s *t*-test followed by Benjamini–Hochberg FDR, in order to adjust *p*-value (adjusted *p*-value < 0.05). We performed the comparison for every miRNA, and ratios between miRNAs and multiple patterns, representing specific transcriptome profiles.

#### For Clinical Correlations

Spearman correlation coefficient test was used to evaluate the main clinical features within sALS subjects, as well as the association between these characteristics and each miRNA (fold change). Furthermore, in order to evaluate the prediction accuracy of each miRNA with respect to the type of disease onset (spinal/bulbar) we used boxplots and ROC curves. ROC curves were plotted with the R package pROC (Robin et al., 2011), while boxplots were produced with the basic R drawing tools.

### MicroRNA Target Analysis

Starting from the results of the DE analysis performed on the two datasets (sRNAs and mRNAs), the relationships between DE miRNAs and DE target genes were investigated through a bioinformatics approach. Their interactions were selected using two databases of experimentally validated bindings (miRtarbase and DIANA-Tarbase). In order to consider the most reliable information about the interactions between the significant miRNAs and their target genes, we selected those bindings that were confirmed in tests with multiple reporters and that were positive at least by Dual Luciferase Reporter Assay, an *in vitro* test that explores the ability of a single miRNA to post-transcriptionally downregulate putative targets through its binding to specific sites within their 3′ UTRs (Clancy et al., 2007; Jin et al., 2013).

### Pathway Analysis

Functional and pathway enrichment analysis of identified DE genes was performed using the Database for Annotation, Visualization and Integrated Discovery (DAVID v6.8^14^) tool. DAVID is a gene functional enrichment program that provides a large series of functional annotation tools and pathway databases (e.g., KEGG, Biocarta, Reactome databases). We determined statistical significance using the one-tailed Fisher’s exact test followed by the Benjamini correction; adjusted *p*-value < 0.05 was set as the threshold value.

## Results

### Study Subjects Characteristics

**Table 1** shows the demographic and clinical characteristics of the sALS and HC groups, at both the discovery and the validation phases. In the discovery phase, the age at blood sample of the HC group was on average about 20 years younger than the sALS patients (*p* = 0.018). In the validation phase, age, or gender did not differ between the patients and controls. Age at disease onset significantly correlated with the progression rate (*r*_s_ = 0.36, *p* = 0.010) and ALSFSRr (*r*_s_ = -045, *p* = 0.001).

**Table 1:** Demographic and clinical characteristics of the study groups.

	ALS	HC
**Discovery phase**	**(*n* = 6)**	**(*n* = 5)**
Gender (number)	4F, 2M	3F, 2M
Age at sample (mean ± SD), years^∗^	69.7 ± 7.6	49.2 ± 14.9^∗^
Age at onset (mean ± SD), years	66.3 ± 6.1	
Clinical signs at onset:		
*Spinal*	5	
*Bulbar*	1	
Disease duration (median, IQR), months	28.6 (13.0)	
Onset-to-diagnosis interval (median, IQR), months	17.3 (6.1)	
ALSFRSr (mean ± SD)	24.7 ± 2.3	
MMT-m (median, IQR)	6.5 (3.6)	
Disease progression rate (median, IQR)	0.8 (0.5)	
Generalization (number)	6 (100%)	
Time to generalization (median, IQR), months	21.8 (16.0)	
**Validation phase**	**(*n* = 50)**	**(*n* = 15)**
Gender (number)	23F, 27M	9F, 6M
Age at sample (mean ± SD), years	64.2 ± 11.0	60.9 ± 5.4
Age at onset (mean ± SD), years	62.5 ± 11.0	
Clinical signs at onset:		
*Spinal*	36	
*Bulbar*	14	
Disease duration (median, IQR), months	18.5 (15.4)	
Onset-to-diagnosis interval (median, IQR), months	12.5 (10.4)	
ALSFRSr (median, IQR)	35.0 (12.0)	
MMT-m (median, IQR)	8.8 (2.0)	
Disease progression rate (median, IQR)	0.7 (0.6)	
Generalization (number)	45 (90%)	
Time to generalization (median, IQR), months	10.7 (12.2)	

*Data are presented as means ± standard deviation (SD), in case of normal distribution, or as median and InterQuartile Ratio (IQR). **^∗^Mann–Whitney U-test**: p = 0.018. F = female; M = male: ALSFRSr, ALS Functional Rating scale; MMT-m, Manual Muscle Testing medium score.*

### Identification of miRNAs and mRNAs Differentially Expressed (DE) in sALS Versus HC

After the discovery experiment performed with HT-NGS methodology, the comparisons of miRNAs expression levels within the study groups revealed **107** mature miRNAs significantly DE between sALS and HC. According to our selection criteria (Liguori et al., 2018), we decided to consider only miRNAs with mean number of reads higher than 25, a Fold Change higher than 2, and a Dispersion Index range of 0–1.6. The **42** miRNAs included in the final list (all downregulated in our sALS population, see **Table 2**) were subjected to qRT-PCR validation as described in the section below. Most of these miRNAs have been shown to be associated with ALS or other neurogenerative diseases (references of the more recent citations in **Table 2**).

**Table 2:** miRNAs/mRNAs interactions.

Transcript_id	Regulation	Published association with ALS or other NDDs	Validated target genes
**let-7a-5p**	Down	**ALS** (Waller et al., 2017b; Taguchi and Wang, 2018)	*HMGA1, MYO1F, PKM, RAB40C*
**let-7d-5p**	Down	**ALS** (Waller et al., 2017a; Taguchi and Wang, 2018)	
**let-7f-5p**	Down	**ALS** (Waller et al., 2017b)	
**let-7g-5p**	Down	**AD** (Mendes-Silva et al., 2016)	
**let-7i-5p**	Down	**ALS** (Waller et al., 2017b; Taguchi and Wang, 2018)	
**miR-103a-3p**	Down	**AD** (Chang et al., 2017)	
**miR-106b-3p**	Down	**^∗^ALS** (Si et al., 2018); **AD** (Guo et al., 2017)	
**miR-128-3p**	Down	**ALS** (Kovanda et al., 2018); **MS** (Vistbakka et al., 2017)	*ABCG1, BAX, CTDSP1, LGALS3*
**miR-130a-3p**	Down		*IFITM1, TGFB1*
**miR-130b-3p**	Down	**ALS** (Taguchi and Wang, 2018)	*SNAI3*
**miR-144-5p**	Down	**ALS** (Raheja et al., 2018)	
**miR-148a-3p**	Down	**ALS** (D’Erchia et al., 2017; Waller et al., 2017b)	*BAX, ITGA5*
**miR-148b-3p**	Down	**MS** (Liguori et al., 2018)	*ITGA5*
**miR-15a-5p**	Down	**ALS** (Recabarren-Leiva and Alarcon, 2018)	*HMGA1, UCP2*
**miR-15b-5p**	Down	**ALS** (Waller et al., 2017a)	
**miR-151a-5p**	Down	**ALS** (Taguchi and Wang, 2018)	*ARHGDIA, OTUB1*
**miR-151b**	Down		
**miR-16-5p**	Down	**ALS** (Waller et al., 2017b); ^∗^(Si et al., 2018)	*ARHGDIA, HDGF, HMGA1, ZYX*
miR-181a-2-3p	Down		
**miR-182-5p**	Down	**ALS** (D’Erchia et al., 2017); **MS** (Liguori et al., 2018)	*FLOT1, NFKBIB, PFN1, SMARCD3*
**miR-183-5p**	Down	**ALS** (D’Erchia et al., 2017)	*PTPA*
**miR-186-5p**	Down	**AD** (Satoh et al., 2015)	*PTTG1*
miR-192-5p	Down	**ALS** (Raheja et al., 2018)	
**miR-22-3p**	Down	**ALS** (Waller et al., 2017b; Kovanda et al., 2018)	*BSG, CD151, LGALS9, PTMS*
**miR-221-3p**	Down	**ALS** (D’Erchia et al., 2017; Di Pietro et al., 2018; Taguchi and Wang, 2018)	*BBC3, GRB10*
**miR-223-3p**	Down	**MS** (Ebrahimkhani et al., 2017)	*HAX1, MYL9*
**miR-23a-3p**	Down	**ALS** (Di Pietro et al., 2018); **MS** (Ebrahimkhani et al., 2017)	*MT2A*
miR-25-3p	Down	**ALS** (Taguchi and Wang, 2018); ^∗^(Si et al., 2018); **MS** (Liguori et al., 2018)	
**miR-26a-5p**	Down	**ALS** (Waller et al., 2017b; Di Pietro et al., 2018; Kovanda et al., 2018; Taguchi and Wang, 2018)	*HMGA1, ITGA5, PHB, PRKCD*
**miR-26b-5p**	Down	**AD** (Chang et al., 2017)	*MIEN1, MT-CO2*
**miR-27b-3p**	Down	**ALS** (Waller et al., 2017b); ^∗^(Si et al., 2018)	*PHB, PINK1*
**miR-28-3p**	Down	**ALS** (Waller et al., 2017b); (Kovanda et al., 2018)	
**miR-30b-5p**	Down	**ALS** (Raheja et al., 2018); **MS** (Ebrahimkhani et al., 2017)	
**miR-30c-5p**	Down	**AD** (Satoh et al., 2015)	*IER2, VIM*
**miR-342-3p**	Down	**AD** (Wang et al., 2017); **MS** (Ebrahimkhani et al., 2017)	
miR-409-3p	Down	**ALS** (D’Erchia et al., 2017); **MS** (Ebrahimkhani et al., 2017)	
**miR-425-5p**	Down	**ALS** (Raheja et al., 2018)	*TACC3*
**miR-451a**	Down	**ALS** (Taguchi and Wang, 2018); **MS** (Ebrahimkhani et al., 2017)	*CDKN2D*
**miR-532-5p**	Down	**MS** (Selmaj et al., 2017)	
**miR-550a-3p**	Down		*MAPK3*
**miR-584-5p**	Down	**ALS** (Kovanda et al., 2018; Taguchi and Wang, 2018)	
**miR-93-5p**	Down	^∗^**ALS** (Si et al., 2018)	*RHOC*

*In the first column (left) are listed those miRNAs that were significantly downregulated in the sALS group compared to HCs (in **bold** the sRNA-Seq confirmed after qRT-PCR). In the last column (right) we report the correspondent experimentally validated (upregulated) genes resulted from our integrated analysis. In the central column (second from the right) are listed some of the references reporting the association between each miRNA and ALS or other neurodegenerative disease. ^∗^Data referred to muscles in animal models (mouse).*

The analysis of mRNA-seq reads identified **4,136** genes that are significantly DE between sALS and HC groups. After filtering for the above three criteria, the expression changes of **1,566** upregulated and **1,761** downregulated genes distinguished sALS patients from HC subjects (total **3,327 genes**, adjusted *p*-value < 0.05) (see also **Supplementary Files**). Interestingly, **12 genes** (***PFN1, TUBA4A, PARK7, SQSTM1, DCTN1, C9orf72, TMEM106B, ALS2, TRPM7, MATR3, SPG11,*** and ***ATXN2***) have been previously associated with ALS (Chia et al., 2018).

### Validation of Significant DE miRNAs in sALS Compared to HC

Five candidate endogenous reference miRNAs (miR-331-3p, miR-423-3p, miR-423-5p, miR-484, and miR-320a) were selected from qRT-PCR blood studies and from miRNA endogenous controls in the TaqMan Advanced miRNA Assays white paper (Applied Biosystem, Thermo Fisher Scientific) (Zheng et al., 2013; Niu et al., 2016). The candidate endogenous reference miRNAs were tested for stable expression across sRNA-Seq data results using the following criteria: (a) high read count in all samples; (b) no intra- and inter-group DE (*p*-value < 0.05). Among the five candidates, Normfinder and GeNorm algorithms determined miR-484 as the best endogenous normalizer and thus we selected it as internal references.

**FIGURE 1:**
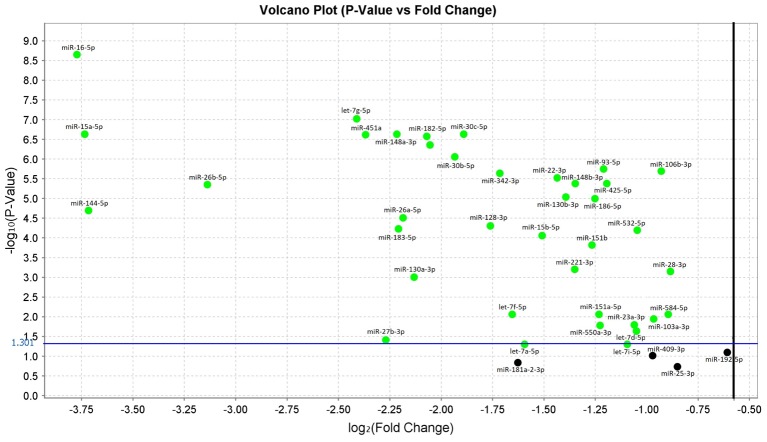
Volcano plot of validated miRNAs. Green dots represent the 38 differentially expressed miRNAs obtained from the comparison between sALS and HC subjects by qRT-PCR (*p* < 0.05). All **black** dots below the blue line did not discriminate sALS from HC. The Y-axis represents the log10 of the *p*-value and the X-axis represents log fold change of miRNA expression in the sALS versus HC.

In the comparison analysis between subjects with sALS and HC, we confirmed that **38** miRNAs were significantly downregulated in sALS patients with *p*-values < 0.05. These miRNAs were: let-7a-5p, let-7d-5p, let-7f-5p, let-7g-5p, let-7i-5p, miR-103a-3p, miR-106b-3p, miR-128-3p, miR-130a-3p, miR-130b-3p, miR-144-5p, miR-148a-3p, miR-148b-3p, miR-15a-5p, miR-15b-5p, miR-151a-5p, miR-151b, miR-16-5p, miR-182-5p, miR-183-5p, miR-186-5p, miR-22-3p, miR-221-3p, miR-223-3p, miR-23a-3p, miR-26a-5p, miR-26b-5p, miR-27b-3p, miR-28-3p, miR-30b-5p, miR-30c-5p, miR-342-3p, miR-425-5p, miR-451a, miR-532-5p, miR-550a-3p, miR-584-5p, miR-93-5p (**Figure 1** and **Supplementary Data**).

Some of the miRNAs that were significantly DE in our study – all downregulated – resulted to be part of five polycistronic miRNA clusters. Specifically, let-7a-5p, let-7d-5p, and let-7f-5p are members of let-7a-1∼let-7d cluster (located in the intergenic region at 9q22.3); miR-16-5p and miR-15a-5p belong to the miR-15a/16-1 cluster (intronic region of DLEU2 gene, locus at 13q14); miR-106b-3p, miR-93-5p, and miR-25-3p are part of the miR-106b∼25 cluster (intronic region of MCM7 gene, located at 7q23.1), although miR-25-3p was not confirmed after the validation step; miR-182-5p and miR-183-5p are members of the miR-182∼96 cluster (intergenic region at 7q32.2) and finally miR-144-5p and miR-451a belong to the miR-144∼451a cluster (located in the intergenic region at 17q11.2).

### Impact of Validated miRNAs Expression on sALS Clinical Measures

**FIGURE 2:**
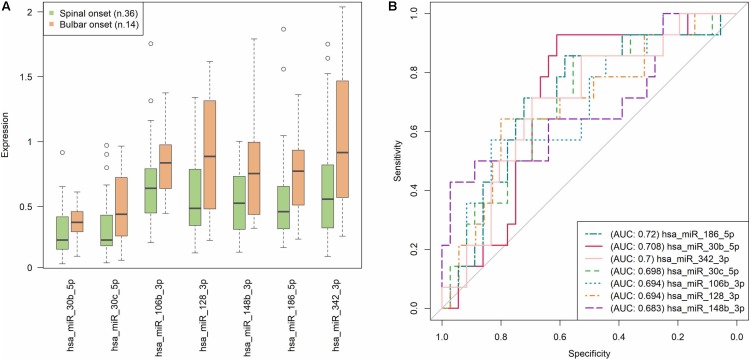
Prediction accuracy of significant miRNAs with respect to the type of disease onset (spinal/bulbar). **(A)** Each boxplot shows the 25^th^ and the 75^th^ percentiles on the bottom and top of the box, the band inside the box is the 50^th^ percentile (the median) and the ends of the whiskers are the minimum and maximum of the data. Outliers are showed as isolated circles. **(B)** ROC curves based on miRNAs relative expression data (as resulted from qRT-PCR analysis). In our sample, the AUCs close to 0.7 suggest that none of the seven miRNAs exerts an exclusive impact on the type of sALS onset, but more likely they act together in order to modulate this phenotypic feature. Note that each value in the ROC curves is considered as a putative numerical threshold for separating the samples in the two onset conditions (spinal/bulbar). Then the sensitivity [true positive rate (TPR)] and Specificity [false positive rate (FPR)] are computed for that threshold. The more the expression values are not-overlapping between the two experimental conditions, the more the ROC curve is near the left and the top axes of the plot, and we obtain an area under the curve (AUC) = 1. The less the conditions are separable, the more the ROC curve approaches the bisector of the plot, with an AUC = 0.5.

Since the most informative clinical data of our sALS patients were longitudinally collected, to identify prognostic markers, e.g., of disease progression, we tested whether the expression of the significant validated miRNAs correlated with any of those markers. Interestingly, the sALS subjects with the most frequent spinal onset (36 out of 50) were characterized by a statistically significant lower expression of miR-106b-3p (*r*_s_ = -0.302, *p* = 0.033), miR-128-3p (*r*_s_ = -0.302, *p* = 0.033), miR-148b-3p (*r*_s_ = -0.284, *p* = 0.046), miR-186-5p (*r*_s_ = -0.342, *p* = 0.015), miR-30b-5p (*r*_s_ = -0.324, *p* = 0.022), miR-30c-5p (*r*_s_ = -0.309, *p* = 0.029), and miR-342-3p (*r*_s_ = -0.312, *p* = 0.028), compared to patients with bulbar onset. **Figure 2A** shows the expression trend of these seven miRNAs grouped by disease onset: their significant overexpression in bulbar onset is clearly visible in the boxplots, even if the distribution of the values are partly overlapping. In **Figure 2B**, the prediction accuracy of each miRNA in discriminating the sALS onset type is represented. The AUC obtained is about 0.7 for all the seven miRNAs; this result means that the correlations between the miRNAs expression and the spinal/bulbar onset is confirmed, although a definite threshold for separating the samples in the two onset conditions (as already seen in the boxplots) is not possible. The similar AUCs obtained also suggest that no miRNA emerges, but the whole group of seven miRNAs synergistically contribute to the different molecular profiles that seem to characterize the phenotypical onset of the disease.

**FIGURE 3:**
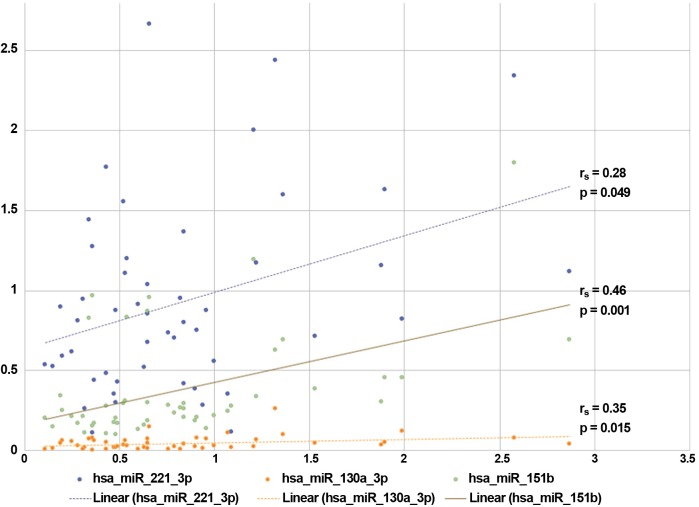
Correlations between miRNAs expression and sALS clinical features. The expressions (fold changes) of three validated miRNAs correlated with the progression rate observed in our sALS population. ^∗^Spearman rank-correlation test: *p* < 0.05.

Among the other parameters, the progression rate positively correlated with the expression of miR-130a-3p, miR-151b and miR-221-3p, as indicated in **Figure 3** (where the correspondent r_s_ and *p*-values are indicated). We also found significant correlations between the expression of (1) miR-27b-3p and the time to disease generalization (*r*_s_ = 0.329, *p* = 0.036), (2) miR-151b and the scores obtained at the ALSFRS-r scale (*r*_s_ = -0.353, *p* = 0.013), and (3) the expression of miR-30b-5p and the MMT-m scores (*r*_s_ = 0.313, *p* = 0.027).

### Identification of Significant Target Genes Possibly Involved in sALS Pathogenesis

The availability of the expression data referred for both miRNAs and mRNAs compounds in our two study groups allowed us to perform an integrated analysis of the expression profiles, which in turn suggested possible functional genetic interactions (miRNA–mRNA) for subsequent experimental validations, thus narrowing down the range of significant candidate genes that resulted from the discovery step.

In fact, the analysis of mRNAs that have been reported as validated targets of the 38 significantly downregulated miRNAs (at least by two algorithms of target prediction, as indicated in Section “Materials and Methods”) returned **162** DE target genes (up or downregulated in our dataset), several in common between different miRNAs; 51 of them were also experimentally validated. Since genes targeted by downregulated miRNAs are expected to be upregulated (due to the reduction of gene silencing effect), we considered experimentally validated interactions only those occurring with **43** genes that fulfilled these criteria (**Table 2** and details in **Supplementary Data**).

**FIGURE 4:**
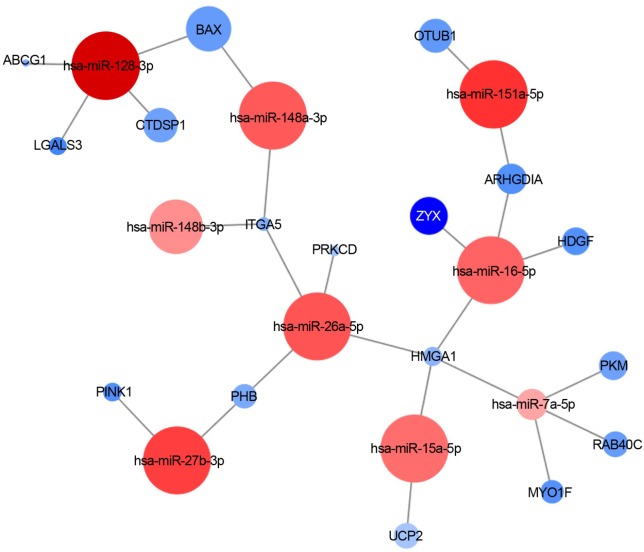
miRNA-target interaction network. An example of experimentally validated miRNA-target gene interactions is visualized as a network (by Cytoscape 3.6.0). Nodes are colored according to the log2 fold change between sALS and HC (**red:** downregulated; **blue:** upregulated), and the node size is proportional to the *p*-value in the DE analysis.

**Figure 4** shows an example of one of the resulted molecular networks outputs that includes nine miRNAs (let-7a-5p, miR-128-3p, miR-148a-3p, miR-148b-3p, miR-15a-5p, miR-151a-5p, miR-16-5p, miR-26a-5p, miR-27b-3p) and 17 target genes (*ABCG1, LGALS3, CTDSP1, BAX, ITGA5, PRKCD, OTUB1, ZYX, ARHGDIA, HDGF, HMGA1, PKM, RAB40C, MYO1F, UCP2, PINK1*, and *PHB*) already shown to be involved in neurodegenerative processes as well as in the immune response functional categories (*see below for details and comments*).

### Implication of Computationally Predicted Molecular Pathways in ALS Pathogenesis

**FIGURE 5:**
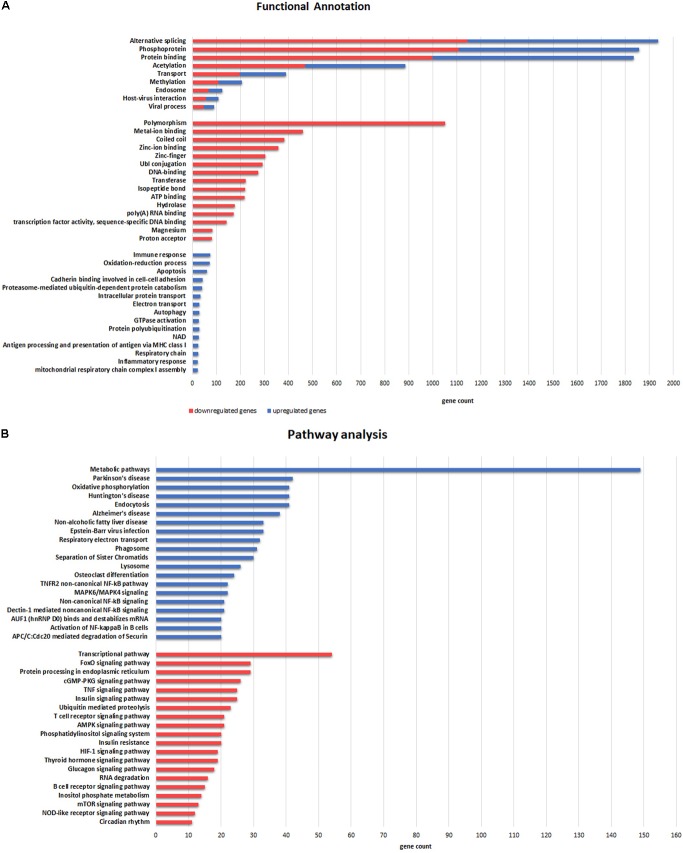
Functional categories **(A)** and Pathway **(B)** enrichment analyses of DE genes. **(A)** Categories enriched in both upregulated (**blue**) and downregulated (**red**) genes are illustrated in the upper part. The more representative categories enriched in downregulated genes are illustrated in the middle section. Categories in the lower part are enriched in upregulated genes. **(B)** Pathways enriched in upregulated (**blue**) and downregulated (**red**) genes are illustrated in the upper and lower part, respectively. The X-axis represents gene counts involved in each functional category and pathway.

A total of **1,936** genes (58% of all DE genes) were significantly enriched (*p*-value = 4,8e^-42^) through an alternative splicing process (**Figure 5A**). Upregulated genes were related to oxidation–reduction and immune response, and mainly located in extracellular exosome, mitochondrion and its inner membrane (cellular component GO-terms). Interestingly, downregulated genes were significantly related to metal binding processes, including zinc, magnesium, and manganese binding. Furthermore, pathway enrichment analysis demonstrated that upregulated genes were significantly involved in metabolic pathways and oxidative phosphorylation (KEGG). In addition, several upregulated genes in ALS samples were associated with other neurodegenerative human disorders such as Parkinson’s disease, Huntington’s disease and AD, whereas downregulated genes were significantly enriched in the transcriptional pathway and the FoxO signaling pathway (**Figure 5B**).

## Discussion

In a well-characterized group of sALS patients we found 38 downregulated miRNAs and 43 experimentally validated upregulated target genes (out of the overall 3,327 DE genes resulted from HT-NGS) in comparison to age-matched HC from the same geographic area. Functional *in vitro* studies are in progress in order to confirm the interactions between the validated miRNAs and the remaining computationally predicted target genes. In our view these findings are a significant contribution to the definition of circulating biomarkers for the diagnosis and monitoring of this devastating disease. In addition, they represent an important step toward the identification of novel candidate genes potentially implicated in the pathogenic mechanisms of ALS.

In the present investigation we were able to evaluate both miRNAs and mRNAs in the same subjects with an extensive and already validated multidisciplinary approach (Liguori et al., 2018) that may be extremely valuable in disorders like ALS, in which the genetic background is usually unavailable. Indeed, the family history ascertained in a limited percentage of ALS patients (fALS) contributed to the identification of inherited mutations in more than 30 genes (Renton et al., 2014; Zou et al., 2017; Chia et al., 2018). However, in the (apparently) sporadic occurrence of the disease only few of these genes (i.e., SOD1, TARDBP, FUS) were found to be implicated. Small sample size of the study groups, misdiagnosis of the affected subjects and incomplete penetrance of genetic risk factors have been evoked to explain the difficulty in identifying the genetic factors contributing to sALS. The risk was found to be substantially lower than expected when estimated through a combined approach of both allele frequency and variant pathogenic prediction (Gibson et al., 2016). Recently, a hexanucleotide expansion in the C9orf72 locus was genotyped both in fALS and sALS patients who more frequently showed a pronounced cognitive impairment compared to those carrying other genetic mutations. This observation suggests that genetic variants may also be associated with ALS phenotypic variability (Shatunov et al., 2010). Modifier genes possibly involved in the prognostic evolution of the disease have also been reported (Brown and Al-Chalabi, 2017; van Es et al., 2017) hence confirming the complexity of the genetic influence in ALS pathogenic definition.

The evidence that miRNAs are implicated in a wide range of fundamental molecular networks as in neurodegeneration (Goodall et al., 2013) has focused the attention on this class of non-coding genes as potential key players in diseases like ALS. In fact, one of the first studies reported a downregulation of a small subset of TDP-43-binding candidate miRNAs in serum and peripheral cell lines of ALS patients (Freischmidt et al., 2013). Homogeneous miRNA alterations were detected in fALS and in asymptomatic mutation carriers, independently from the affected genes (Freischmidt et al., 2014). On the contrary, the observation of a highly heterogeneous miRNA profile in sALS indicates a multiform molecular etiology in the sporadic occurrence of the disease (Freischmidt et al., 2015; Prudencio et al., 2015). Also a different expression of several miRNAs found in ALS-FTD further supports the existence of many patterns related to the main causative genes and it suggests that different mutations may be characterized by subtype-specific miRNA signatures (Gascon and Gao, 2014). Several other studies have been carried out to identify suggestive miRNAs associated with sALS in CSF as well as in other biological fluids (Waller et al., 2017a,b; Matamala et al., 2018; Raheja et al., 2018; Vejux et al., 2018; Vrabec et al., 2018) and in skeletal muscles (Di Pietro et al., 2018; Kovanda et al., 2018) of affected individuals. Based on computational prediction of miRNAs that targeted significant mRNAs available in public repository (GEO), new strategies that used both the transcriptomic compounds (miRNAs and mRNas) to delineate the complete picture of ALS showed to be very effective (Mitropoulos et al., 2018; Taguchi and Wang, 2018).

Using our sALS cohort data (all but six negative for the most common ALS-associated genes) we were able to identify **38** downregulated miRNAs significantly associated with sALS (data confirmed with/without the mutated cases). Our study confirmed the possible pathogenic involvement of these miRNAs, since most of them were previously reported in ALS (let-7a-5p, let-7d-5p, let-7f-5p, let-7i-5p, miR-128-3p, miR-130b-3p, miR-144-5p, miR-148a-3p, miR-15a-5p, miR-15b-5p, miR-151a-5p, miR-16-5p, miR-182-5p, miR-183-5p, miR-22-3p, miR-221-3p, miR-23a-3p, miR-26a-5p, miR-27b-3p, miR-28-3p, miR-30b-5p, miR-425-5p, miR-451a, and miR-584-5p) and/or other neurodegenerative syndromes, especially Alzheimer’s Dementia (let-7a-5p, let-7d-5p, let-7f-5p, let-7g-5p, let-7i-5p, miR-103a-3p, miR-106b-3p, miR-144-5p, miR-148a-3p, miR-15a-5p, miR-15b-5p, miR-186-5p, miR-22-3p, miR-26a-5p, miR-26b-5p, miR-28-3p, miR-30c-5p, miR-342-3p, and miR-425-5p, see **Table 2** for references). Among the remaining, miR-223-3p (never associated with ALS) was reported to positively impact the neuronal activity, since its overexpression induced a neuroprotective effect by targeting the glutamate receptors (Harraz et al., 2012). Therefore it is reasonable to hypothesize that its downregulation (as documented here) may promote the neuronal cell death, leading to the irreversible neurodegenerative processes observed in ALS.

We also found that some of the significant miRNAs belong to five different clusters. This observation supports evolutionary as well as functional implications in the pathogenic processes in which they were involved (Altuvia et al., 2005). *In vitro* functional studies will allow future testing of related hypotheses, e.g., the possibility that all or some of these miRNAs may act in synergy on the target genes. In our investigation, however, downregulation of both miR-15a/16-1 and miR-106b∼25 clusters did not correlate with the expression of their respective host genes (DLEU2 and MCM7), as resulted in the analysis of our RNA-Seq data. The discordance in the expression between intronic miRNA-host mRNA pairs could be explained by the occurrence of an alternative splicing event in the region of clustered miRNAs (Ramalingam et al., 2014). Finally, it is worthy to mention that the downregulation of miR-106b-25 cluster seems to play an important role in the apoptosis induced by endoplasmic reticulum stress strongly associated with the disease progression and the motoneuron degeneration in the ALS animal model (Gupta et al., 2012).

The analysis of DE genes (mRNAs) confirmed the importance of 12 genes *(PFN1, TUBA4A, PARK7, SQSTM1, DCTN1, C9orf72, TMEM106B, ALS2, TRPM7, MATR3, SPG11*, and *ATXN2)* whose mutations have been already identified as causative of sALS/fALS (Renton et al., 2014; Morgan and Orrell, 2016; Al-Chalabi et al., 2017; Brown and Al-Chalabi, 2017; van Es et al., 2017; Zou et al., 2017; Chia et al., 2018). In future analysis we will perform exomes sequencing to check the presence of pathogenic mutations in the genes of recruited patients. However, by looking at the upregulated and downregulated DE genes we were able to delineate a pathogenic landscape where several molecular functions are evoked. In particular, the downregulated genes are involved in the molecular bindings of ions (i.e., metal ions, zinc, and magnesium) and in some ions-related functions. The upregulated genes (less numerous) are implicated in the immune response, oxidation–reduction processes, and apoptosis. Taken together, our data confirm the idea that dysfunctions in these networks might contribute to the pathogenesis of the disease, as already reported by functional *in vitro* studies (Bourassa et al., 2014; Alvarez-Zaldiernas et al., 2016; Peters et al., 2017). Furthermore, the downregulated 9-miRNAs system (**Figure 4**) evoked significant upregulation of genes found to be associated with the pathogenic background of ALS. In particular, the gene coding for galactin-3 (*LGALS3*) (Chen et al., 2014), a protein with controversial functions in neuroinflammation and possibly involved in the deposition of intracellular pathological aggregation of gamma-synuclein was observed in ALS (Peters et al., 2015). As confirmation, high levels of plasma galactin-3 have been reported in Chinese ALS patients (Yan et al., 2016). Furthermore, our data strongly support the mitochondrial pathogenic hypothesis of the disease, as indicated by the significant differences in the expressions of target genes like *BAX* (a pro-apoptotic marker interacting with 14-3-3 protein and responsible for increased cell death, in presence of mutant SOD1) (Park et al., 2017), *UCP2* (its overexpression has been demonstrated to further deteriorate mitochondrial dysfunctions and the ALS progression in animal models of the disease) (Peixoto et al., 2013), *PRKCD* (activation of the gene seems to modulate mitochondrial-induced apoptosis) (Dave et al., 2005) and *PINK-1* (ablation of this gene rescued the mitochondrial axonal transports defective in complex model of ALS) (Moller et al., 2017).

**FIGURE 6:**
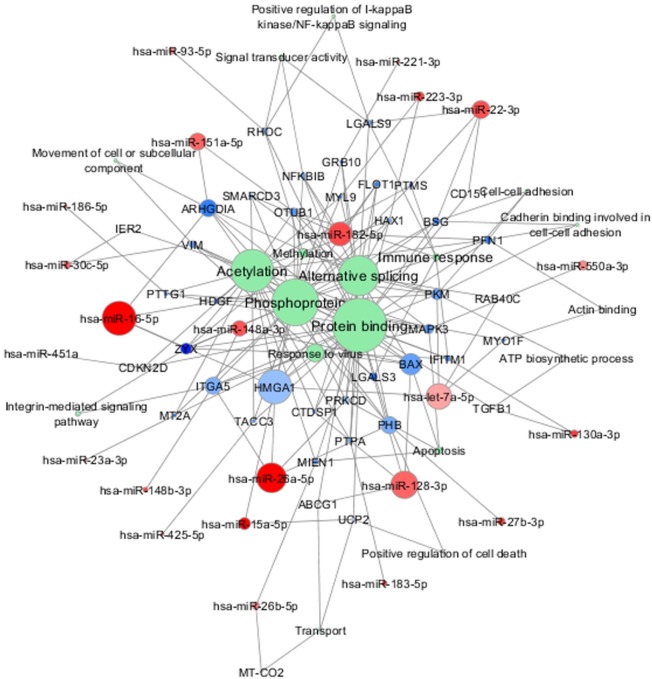
Combined molecular analysis in sALS. Functional annotations of experimentally validated target genes together with their miRNAs are visualized as a network (Cytoscape 3.6.0). Each node size is proportional to the degree of the node (connectivity). **Green** nodes correspond to functional annotations obtained by functional enrichment analysis (DAVID tool). Color gradient intensity of miRNA and target nodes correlates with upregulated (**blue**) or downregulated (**red**) expression levels.

Incomplete expression overlap was found between the validated miRNAs and their target genes, as already reported (Freiesleben et al., 2016). *In vitro* studies investigating the impact of the significant miRNAs in the expressions of the complete panel of their target genes may clarify this aspect. Meanwhile, in **Figure 6** we summarized the network composed by the 38 validated miRNAs, their 43 validated target genes and the common functional annotations between them, in which the above-mentioned genes represent the connection nodes. In this representation we highlighted other genes in the intermodal connections that may be worthy of further investigations, like *TGF-beta1*. This gene seems in fact to be involved in ALS pathogenesis, as enhanced secretion of TGF-beta1 was observed in reactive astrocyte that greatly contributed to motor neuron protein aggregation and neurite degeneration, e.g., *via* the inhibition of cellular autophagy, independently from SOD1 wild-type/mutation status (Tripathi et al., 2017). We also noted that other genes like *MT-CO2*, *MT2A*, and *ABCG1* are placed in specific nodes of our molecular networks, thus suggesting that they may play important role/s in ALS, as it has been already reported in other neurodegenerative diseases like AD (Hayashi et al., 2006; Beecham et al., 2014; Lunnon et al., 2017).

**FIGURE 7:**
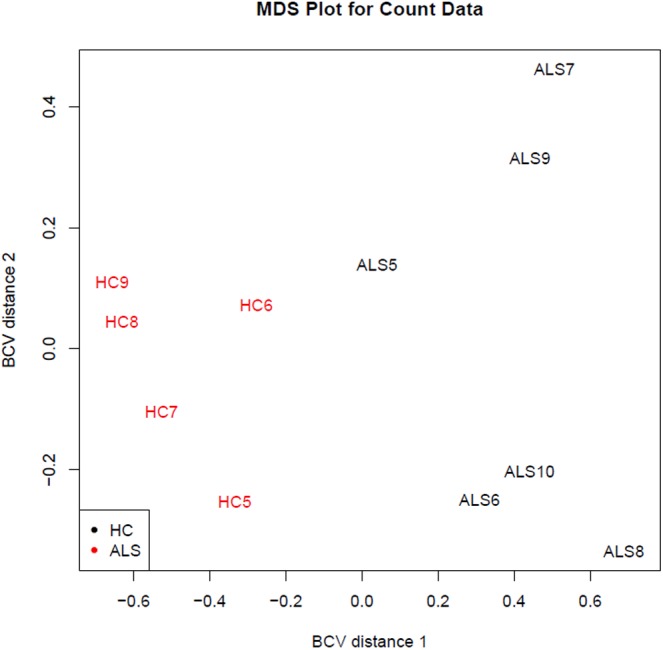
Distribution of the study samples by sRNA-seq output (discovery phase). The sRNA-Seq data were explored by generating multi-dimensional scaling (MDS) plots. This visualizes the differences between the expression profiles of different samples in two dimensions using the biological coefficient of variation (BCV). The MDS plot shows clear separation of the sALS vs. HC samples.

One limitation in our study is a possible bias related to the age of HC subjects, significantly younger than the sALS individuals, in the discovery phase. This occurred because of the difficulty in finding age-matched subjects without any comorbidity, i.e., cardiovascular risk factors, at the very early phase of the investigation. However, as we have shown, this gap was filled out in the validation step. Since 38 out of 42 miRNAs (90.5%) were confirmed in the independent validation sample, we believe that the group difference in age at the discovery phase did not impact the identification of the final set of genetic data. Another limitation is the relatively small number of subjects submitted to the NGS analysis. However, our study numbers are in line with others using the same time-consuming and expensive approach. Most importantly, the analysis of both the raw data of sRNA-Seq and RNA-Seq showed that we were able to significantly discriminate the two populations (as represented in the **Figure 7**).

In the last few decades, neurodegenerative diseases have become a major challenge for the National Public Health systems due to the increasing social and economic implications of these diseases. Therefore, the discovery of circulating markers in diseases like ALS will be of great value also in the perspective of finding more effective therapeutic tools.

Our comprehensive investigation of combined miRNAs/mRNAs profiles in sALS revealed a complex molecular network in which miRNAs and target genes connect to several functional categories. The results also suggest the presence of peculiar molecular prognostic traits, associated with the bulbar/spinal onset and the slope of clinical progression. If these results will be confirmed in a larger study, then changes in these circulating biomarkers may represent critical early prognostic signs of clinical deterioration and be extremely relevant in guiding future therapeutic efforts.

## Data Availability

The raw data supporting the conclusions of this manuscript will be made available by the authors, without undue reservation, to any qualified research.

## Author Contributions

ML and ILS contributed conception and design of the study. NN performed the molecular analysis and the subsequent data elaboration. AI coordinated the clinical evaluations of the patients and the recruitments of all subjects with the contributions of ED’E, AS, and ED. AC and FL performed the bioinformatics and statistical analysis. ML wrote the first draft of the manuscript with the contribution of ILS and AI (clinical section), AC and FL (statistical and bioinformatics section), NN (molecular data). All authors contributed to the manuscript revision and read and approved the submitted version.

## Conflict of Interest Statement

The authors declare that the research was conducted in the absence of any commercial or financial relationships that could be construed as a potential conflict of interest.

## Footnotes

1 https://www.wma.net/policies-post/wma-declaration-of-helsinki-ethical-principles-for-medical-research-involving-human-subjects

2 http://www.bioinformatics.babraham.ac.uk/projects/fastqc

3 http://www.mirbase.org

4 http://vega.archive.ensembl.org/info/about/gene_and_transcript_types.html

5 https://www.ensembl.org/index.html

6 https://www.ncbi.nlm.nih.gov/refseq

7 http://pirnabank.ibab.ac.in

8 http://gtrnadb.ucsc.edu

9 http://www.genenames.org

10 https://www.mdc-berlin.de/media/16193

11 https://bioconductor.org/packages/release/bioc/html/edgeR.html

12 https://bioconductor.org/packages/3.7/bioc/html/DESeq2.html

13 https://bioconductor.org/packages/3.7/bioc/html/limma.html

14 http://david.abcc.ncifcrf.gov

## Abbreviations

AD: Alzheimer’s disease
ALS: amyotrophic lateral sclerosis
ALSFRS-r: revised ALS Functional Rating scale
CSF: cerebrospinal fluid
DE: differential expression
fALS: familial amyotrophic lateral sclerosis
FTD: frontotemporal dementia
HC: healthy control
HT-NGS: high-throughput next-generation sequencing
miRNA: microRNA
MMT: manual muscle testing
mRNA: messenger RNA
nc-RNA: non-coding RNA
ncRNAdb: non-coding RNA database
NF: neurofilament
ODI: onset-diagnosis interval
RNA-Seq: RNA sequencing
sALS: sporadic amyotrophic lateral sclerosis
sRNA: smallRNA
sRNA-Seq: smallRNA sequencing
