# Corrigendum: Dysregulation of MicroRNAs and Target Genes Networks in Peripheral Blood of Patients With Sporadic Amyotrophic Lateral Sclerosis

**DOI:** 10.3389/fnmol.2018.00488

**Published:** 2019-01-10

**Authors:** Maria Liguori, Nicoletta Nuzziello, Alessandro Introna, Arianna Consiglio, Flavio Licciulli, Eustachio D'Errico, Antonio Scarafino, Eugenio Distaso, Isabella L. Simone

**Affiliations:** ^1^National Research Council, Institute of Biomedical Technologies, Bari Unit, Bari, Italy; ^2^Department of Basic Sciences, Neurosciences and Sense Organs, University of Bari, Bari, Italy

**Keywords:** sporadic amyotrophic lateral sclerosis, microRNA, target genes, peripheral blood markers, high throughput next-generation sequencing (HT-NGS), clinical parameters, bioinformatics, pathway analysis

In the original article, there was a mistake in Figure 1 as published. We used the Volcano Plot, which was automatically generated using the Expression Suite v1.0.3 software (Thermo Fisher Scientific). This software visualizes the *p*-values and fold change values after each qRT-PCR run. A log2 (Fold Change) greater than or equal to one, is the standard boundary field.

Therefore, targets with a fold less than the lower boundary were automatically colored in green. However, as stated in the article, according to published guidelines, we validated the miRNAs with *p*-values < 0.05 (dot over the blue line) independently from their fold change. We have therefore adjusted the representation in the figure to avoid confusion.

The corrected Figure 1 appears below. The authors apologize for this error and state that this does not change the scientific conclusions of the article in any way. The original article has been updated.

**Figure 1:**
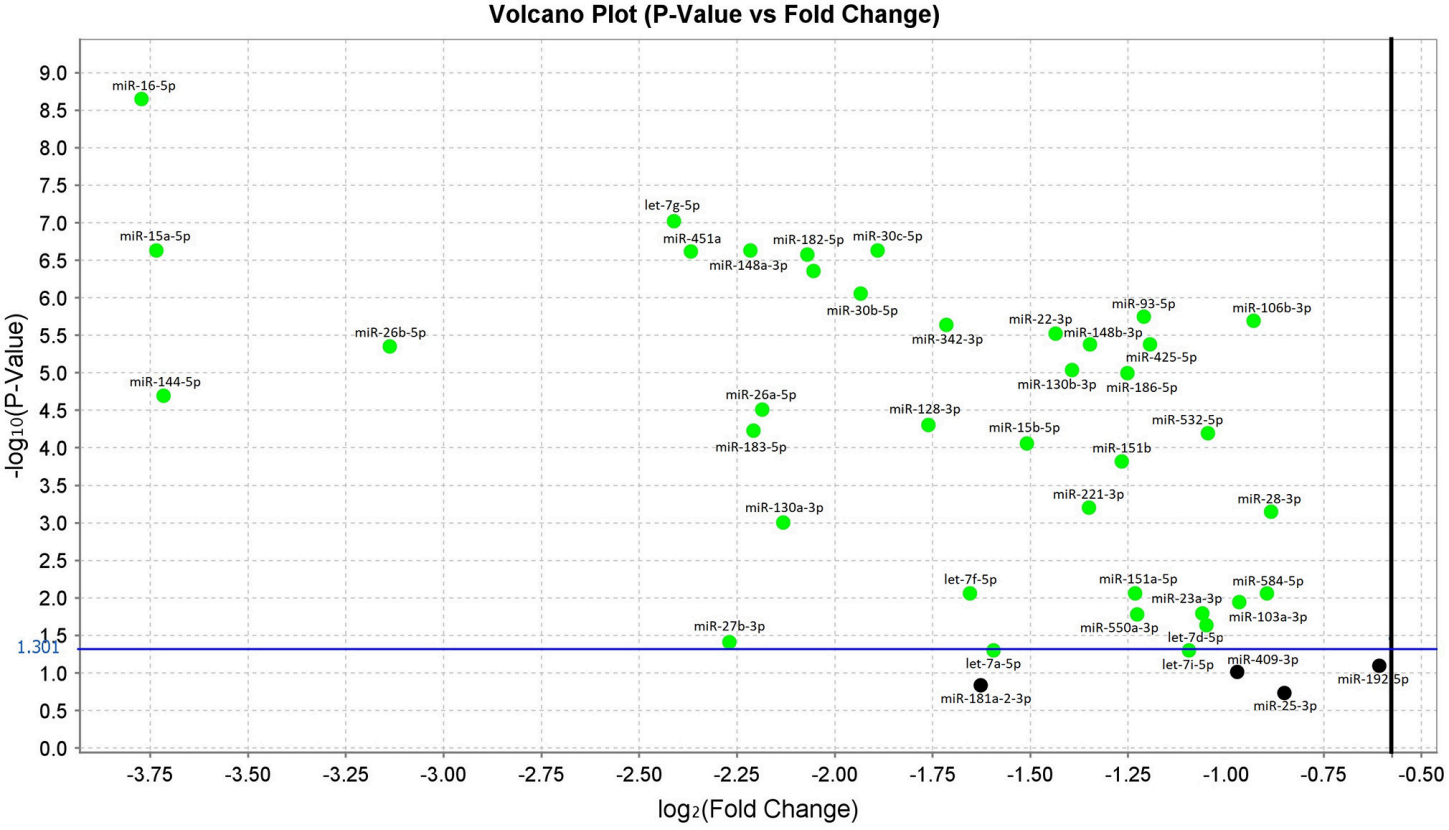
Volcano plot of validated miRNAs. Green dots represent the 38 differentially expressed miRNAs obtained from the comparison between sALS and HC subjects by qRT-PCR (*p* < 0.05). All black dots below the blue line did not discriminate sALS from HC. The Y-axis represents the log10 of the *p*-value and the X-axis represents log fold change of miRNA expression in the sALS versus HC.

